# Effects of Compaction Velocity on the Sinterability of Al-Fe-Cr-Ti PM Alloy

**DOI:** 10.3390/ma12183005

**Published:** 2019-09-16

**Authors:** Xianjie Yuan, Xuanhui Qu, Haiqing Yin, Zhenwei Yan, Zhaojun Tan

**Affiliations:** 1 Colleage Mechanic, North China University of Water Resources and Electric Power, Zhengzhou 450045, China; 2 Institute for Advanced Material and Technology, University of Science and Technology Beijing, Beijing 100083, China

**Keywords:** powder metallurgy, high velocity compaction, aluminum alloy, sintering

## Abstract

In this research, the effects of the compaction velocity on the sinterability of the Al–Fe–Cr–Ti powder metallurgy (PM) alloy by high velocity compaction were investigated. The Al–Fe–Cr–Ti alloy powder was compacted with different velocities by high velocity compaction and then sintered under a flow of high pure (99.999 wt%) nitrogen gas. Results indicated that both the sintered density and mechanical properties increased with increasing compaction velocity. By increasing the compaction velocity, the shrinkage of the sintered samples decreased. A maximum sintered density of 2.85 gcm^−3^ (relative density is 98%) was obtained when the compaction velocity was 9.4 ms^−1^. The radial and axial shrinkage were controlled to less than 1% at a compaction velocity of 9.4 ms^−1^. At a compaction velocity of 9.4 ms^−1^, sintered compacts with an ultimate tensile strength of 222 MPa and a yield strength of 160 MPa were achieved. The maximum elongation was observed to be 2.6%. The enhanced tensile properties of the Al–Fe–Cr–Ti alloy were mainly due to particle boundary strengthening.

## 1. Introduction

As energy shortages and environmental pollution become increasingly serious, there is an increasing desire for lightweight materials that can provide energy savings and environmental protection in the automobile industry. One option that has become progressively more attractive is that of aluminum powder metallurgy (PM) [1]. A uniform microstructure is difficult to achieve by conventional die casting techniques because of the limited solubility of transition metals (TMS), such as Cr, Fe, Ti, and Zr in the Al matrix and the low cooling rate during solidification. On the other hand, the PM process for the fabrication of aluminum alloys allows one to tailor the composition of the alloy elements without segregation [2]. The field of aluminum PM is of particular interest, as the implementation of this technology to produce vehicle components could offer a combination of weight savings because of the low density of aluminum and material savings via aluminum PM near net shape processing attributes [3]. In addition, the aluminum PM also has an advantage in subsequent processing, for example, in rust protection and demagnetization fields. Press-and-sinter aluminum PM is an ever-growing industry because of the cost savings associated with its high production rates and net shape capabilities coupled with the advantageous strength-to-weight ratio inherent to aluminum alloys [4].

Studies on press-and-sinter aluminum alloys have focused on sintering and expanding the scope of its automotive applications. Al alloy powder is difficult to sinter because of the stable aluminum oxide film covering the powder particles. This film presents a formidable barrier to successful sintering. Several mechanisms for aluminum oxide disruption or reaction have been proposed, including [5]: (1) The higher the compaction pressure, the more effective the sintering operation is [6]; (2) adding elements that can aid the decomposition of the oxide layer, such as Mg [2,7]; (3) adding elements to create a liquid phase during sintering that penetrates into the powder and improves the adhesion of the powder, such as Si [8], Sn [9], Ag [10], and Cu [11]; and (4) the sintering of the aluminum alloy is enhanced under a nitrogen atmosphere [12]. Significant effort has been put into developing aluminum PM materials that can be used to expand the scope of automotive applications [13], such as Al–Zn–Mg–Cu [14], Al–Si [15], Al–TMS (transition metals) [16], Al–Cu–Mg [3], and Al–Ni–Mg– (Cu) [17]. The Al–Fe–Cr–Ti alloy contains transition metals, such as Cr, Fe, and Ti, which are slow to diffuse in solid aluminum and whose alloying elements exhibit low solid solubility in aluminum [18,19]. It is easy to cause segregation when producing this alloy with conventional die casting techniques. However, rapidly solidified powder has exhibited finely divided and uniformly dispersed pro-eutectic crystals that could result in reduced segregation [20]. Meanwhile, high velocity compaction (HVC) is an efficient technology for preparing high density PM components [21]. HVC has greatly enhanced the performance of green bodies [22,23,24,25,26] with various advantages, such as low ejection force, uniform density distribution, low spring back, and high density. The process of HVC was simulated using DEM (discrete element method), and the change of porosity in HVC was captured [27]. High impact energy was achieved by modifying the existing equipment, and the hydraulic control system was developed to implement automatic control of the energy produced from the disc springs [28]. Zhang et al. proposed a machine-learning approach based on materials informatics to predict the green density of compacts using relevant material descriptors, including chemical composition, powder properties, and compaction energy [29]. During the HVC, rapid hammer impacts on powder particles produced very high pressure [26], and the contact area among the powders increased. Higher pressure is more efficient in destroying the aluminum oxide layer, as the oxide film fracture facilitates Al–Fe–Cr–Ti alloy sintering. Therefore, HVC not only generates a high density of the green material but also helps it to sinter. Thus, it is very important to use HVC to compact and sinter the powder of this Al-based alloy.

It has been shown in our previous research that the compaction of Al alloy powder using HVC could result in excellent green performance [26]. The objective of this work is to undertake a comprehensive study on the sintering of an Al–Fe–Cr–Ti alloy. Aluminum alloy powder was pressed by HVC with a single impact, using different compaction velocities, and then sintered under a flow of high pure (99.999 wt%) nitrogen gas. This alloy has been characterized based on its sintered density, shrinkage, and tensile properties. In particular, the microstructural evolution and strengthening mechanisms of the sintered sample were studied.

## 2. Materials and Methods

### 2.1. Al–Fe–Cr–Ti Alloy Powder

**Figure 1:**
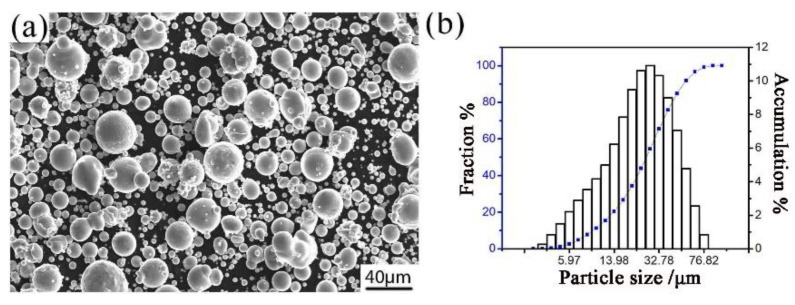
The image (**a**) and particle size distribution (**b**) of the Al–Fe–Cr–Ti alloy powder.

Gas atomized Al–Fe–Cr–Ti alloy powder was produced in the Central South University and was used as the raw material for this research. The chemical composition of the aluminum alloy powder is shown in Table 1. The morphology of the powder is shown in Figure 1a, which shows that particles in the powder exhibit a nearly spherical shape. Figure 1b shows the particle size distribution of the atomized aluminum alloy powders. The median diameter of the powder particle is 25 μm.

### 2.2. High Velocity Compaction

The HVC process for Al alloy was researched and expounded in our previous work [26]. HVC is a process that is carried out under normal temperature and natural air. During HVC, the compaction energy is proportional to the stroke length of the hammer. The compaction energy was calculated based on the formula, as follows:

(1) E=Fh

where E is the compaction energy, F is the force applied to the hammer by the hydraulic system, and h is the stroke length (the distance between the starting position and impact position). For the HYP35-2 HVC machine; F is 26.5 kN. Since the kinetic energy is proportional to the square of the velocity, the impact energy is equal to the kinetic energy. Hence, the velocity can be calculated based on the formula, as follows:

(2) $$E\;={\;\mathrm{mv}}^{2}/2$$

(3) $$v=\sqrt{2E/m}$$

(4) $$v=\sqrt{2\mathrm{Fh}/m}$$

where v is the compaction velocity, m/s; and m is the weight of the hammer (i.e., 42 kg). E can be obtained from Equation (1). The relationship between stroke length and velocity is shown in Table 2.

In our previous experiment, we introduced a comparison between single impacts and multiple impacts. The single impact showed to achieve a higher green density for the Al alloy. Therefore, green samples from a single impact were selected for the sintering experiment. During HVC, the hammer and upper punch (driven by hydraulic power), traveling at speeds of 5.0–9.4 ms^−1^, impacted the powder and applied a very large force to the powder to produce densification. The die-wall lubricant adopted zinc stearate dissolved in acetone before powder filling in the die to decrease the friction between the particles and the die wall and facilitate the ejection of the samples. The die diameter was 25 mm, and the filling height was kept at 15 mm. A total of five specimens were produced in each group at each compaction velocity. Cylinder samples with a diameter of 25 mm and a height of 8.4–9.5 mm were obtained.

### 2.3. Sintering

**Figure 2:**
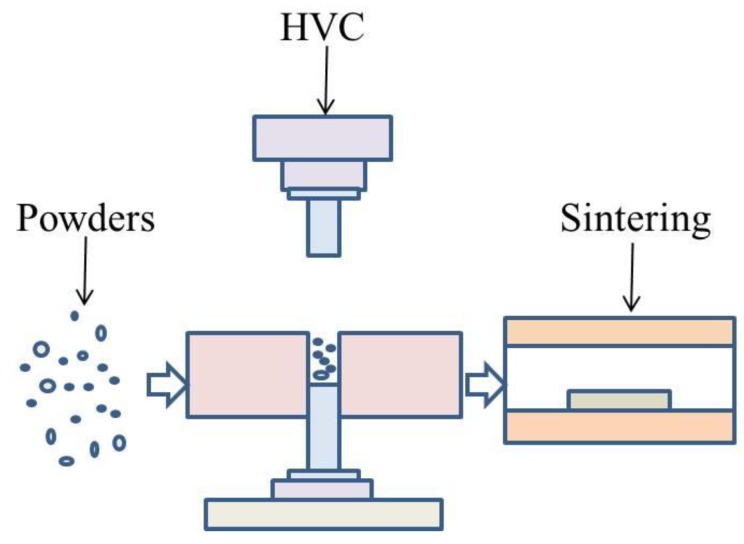
Schematic drawing of the manufacturing process.

Aluminum alloy powder was compacted by HVC and formed the green compacts, which were sintered. The schematic drawing of the manufacturing process is illustrated in Figure 2. The green compacts with different compaction velocities were sintered under a flow of high pure (99.999 wt%) nitrogen gas in a tube furnace. The aluminum alloy was rapidly heated during the sintering process, and the heating rate was increased by 10 °C/min because of the restriction of the furnace. However, during the HVC, a very large impact force was applied to the powder, which produced a great deal of residual stress in the green material. The rapid elimination of residual stress was harmful for the sinterability. Therefore, during the process of low temperature heating, one should choose a slower rate of heating and insulation in order to release the residual stress. The furnace was programmed to heat with a rate of 4 °C min^−1^ to 315 °C, holding for 60 min to reduce the influence of residual stress. Following this, the specimens were heated to a set point of 640 °C, with a rate of 10 °C/min, and isothermally held for 60 min. Then they were kept in the furnace and allowed to cool to ambient temperature. This sintering process was experimentally optimized.

### 2.4. Performance Measurement

A differential scanning calorimetry (DSC) method (Netzsch STA 449C, Selb, Germany) was conducted in a temperature range of 40–900 °C at a constant heating rate of 10 °C/min to measure the temperature range of the decomposition of the quasicrystal phase.

The sintered density was obtained with the Archimedean principle according to ASTM B962-08. The phase identification of the sintered samples was performed using a Siemens D5000 X-ray diffractometer (XRD, Siemens, Munich, Germany) with a Cu Ka radiation source operating at a voltage of 40 kV, with a current of 40 mA, and a continuous scanning rate of 5 °C /min. The microstructures of the sintered samples were examined by a JSM-6510 scanning electron microscope (SEM, JEOL, Tokyo, Japan). The morphology of the powder and the sintered sample was observed by a field emission scan electron microscope (FESEM, ZEISS ULTRA55, ZEISS, Oberkochen, Germany) equipped with energy dispersive spectroscopy (EDS) and a transmission electron microscope (TEM, FEI Tecnai G2 F20 S-Twin, FEI, Hillsborough, OR, USA). Samples for TEM observation were prepared by the preparation technique involving cutting of 3 mm disks, grinding, dimpling, and finally ion-milling using Ar gas ions at 5 kV at an angle of 4° until perforation occurred.

All of the samples for the tensile testing were cut from the sintered specimens and polished into dog-bone-shaped specimens with a gauge dimension of 15 × 3 × 2.5 mm^3^. At room temperature, the tensile tests were performed using an Instron universal testing machine at a constant cross-head speed of 0.5 mm/min. For each compaction velocity, the average value of the three specimens was used to evaluate the strength and elongation. The fracture surfaces of the sintered samples were also investigated by SEM.

### 2.5. The Theoretical Density

Densities were expressed as a percentage of the theoretical density ρ_TD_, which was estimated using a weighted average of the elemental densities using Equation (5) [5]:

(5) $$\rho_{\mathrm{TD}}=n/\sum_{i=1}^{t}(n_{i}/\rho_{i}){\;\rho }_{\mathrm{TD}}=n/\sum_{i=1}^{t}(n_{i}/\rho_{i})$$

where $n=\sum_{i=1}^{t}n_{i}$ and $\rho_{i}$ is the density of element i, and $n_{i}$ is the wt% of element i divided by the atomic weight of element i. This estimation did not consider the proportions and densities of the constituents present after sintering. As the density of the Al, Fe, Cr, Ti element are 2.70 gcm^−3^, 7.88 gcm^−3^, 7.19 gcm^−3^, 4.54 gcm^−3^, respectively, the theoretical density of the Al–Fe–Cr–Ti alloy was 2.92 gcm^−3^.

## 3. Results

### 3.1. Characterization of Al-Fe-Cr-Ti Alloy Powder

The size and microstructure of the particles obtained by this method of atomization are different because of their different particle sizes with different cooling rates. In the gas atomization process, the smaller particles quickly become solid owing to their faster cooling rate. Therefore, their microstructures are fine and uniform. The larger particles slowly become solid because of their slower cooling rate. Therefore, their microstructures are composed of metal compounds [30].

**Figure 3:**
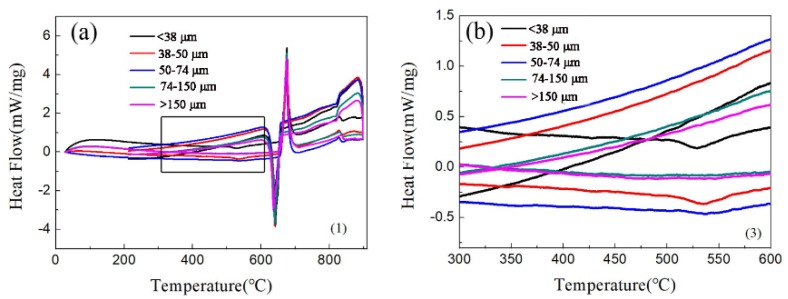
The DSC of aluminum alloy powder:(**a**) full scan from 10 to 90 °C; (**b**) enlargement of the 300 to 600 °C section.

Figure 3 is the DSC of the aluminum alloy powder. Figure 3a shows a major endothermic peak at ~655 °C, which demonstrates that the melting point of the alloy is 655 °C. The experimental sintering temperature was 640 °C. Therefore, it was not liquid phase sintering. At 530 °C, particles with a particle size of fewer than 50 μm have an exothermic peak, as shown in Figure 3b. Figure 3b shows that there is a Q-phase decomposition at 530 °C in the small particles. During the sintering process, the Q-phase decomposes because of the high temperature, and then the phase will increase with an increase in temperature. Galano et al. [31] found that the Q-phase decomposition temperature of Al-based matrix/γ-Al_2_O_3_ nanocomposites varied from 503 °C to 551.6 °C with the powder milling time. The different values reported for the decomposition temperature could be related to the different process, and solute contents, such as the Ti-content retained in the Al matrix [18].

**Figure 4:**
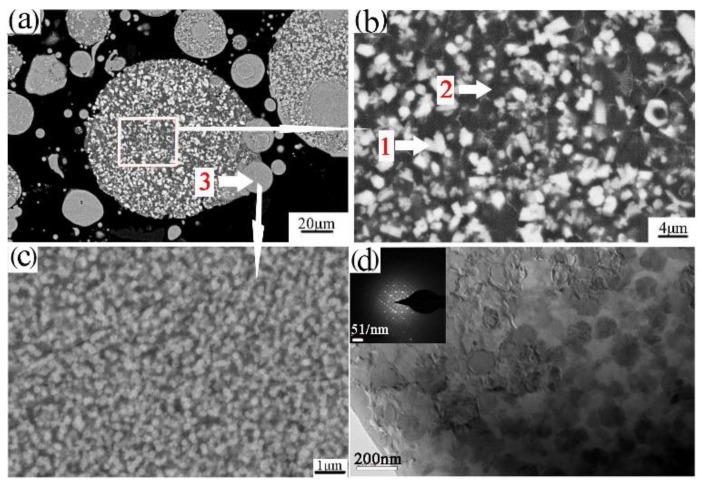
The SEM (scanning electron microscope)/BSE microstructure of the transverse section of atomized powder (**a,b**); FESEM (field emission scan electron microscope)/BSE micrographs of the fine powder particle (**c**); bright field TEM (transmission electron microscope)/BF micrographs of the fine powder particles (**d**).

**Figure 5:**
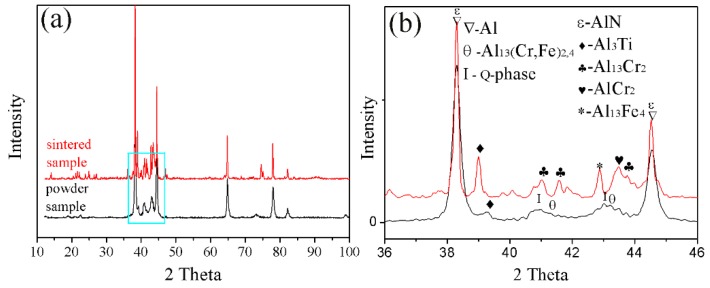
XRD pattern of the powder and sintered samples: (**a**) full scan from 10 to 90°. (**b**) enlargement of the 36 to 46° section.

Powder particles and 93% of the epoxy resin and 7% of the ethylenediamine solution were mixed and then injected into the plastic pipe, with 24 h of rest, to obtain the sample for grinding. The upper surface of the sample was ground smooth to observe the microstructure, as shown in Figure 4a,b. Figure 4a,b shows that the powder was composed of the white phase 1, black phase 2, and phase 3. Further EDS determined phase 1, phase 2, and phase 3 of the composition, as shown in Table 3. Phase 1 is rich in the alloying elements Cr, Fe. Phase 2 is rich in the alloying element Ti. Phase 3 of the fine powder particle is consistent with the composition of the aluminum alloy. The FESEM of the fine powder particles showed a uniform dispersion of the nanophases on the Al matrix, as shown in Figure 4c. The phase composition of the powders was further analyzed by XRD, as shown in Figure 5a. XRD analysis revealed that the phases of the powder are the Al_3_Ti, Al1_3_(Cr, Fe)_2,4_, and quasicrystalline phase (Q-phase). The existence of Q-phase was found, as further confirmed by TEM and shown in Figure 4d.

### 3.2. Sintered Density

**Figure 6:**
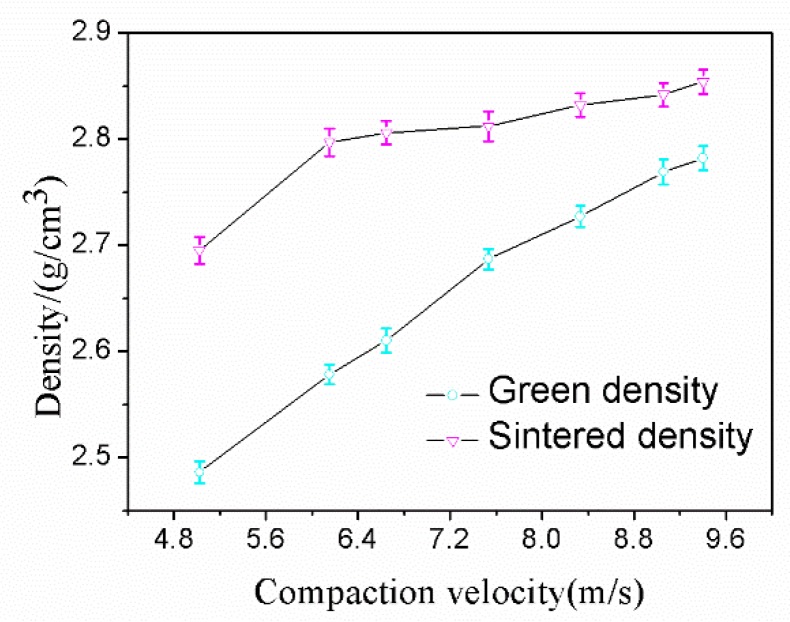
Density versus compaction velocity.

Aluminum alloy powder was pressed by a single impact under a range of compaction velocities from 5.0 to 9.4 ms^−1^ and sintered with the sintering process established for the alloy in this research. The data from the green density and sintered density measurement experiments are shown in Figure 6. From Figure 6, it can be seen that the green density and sintered density both increased with an increasing compaction velocity. For the sintered density, a considerable turning point was found for samples at a compaction velocity of 5.6 ms^−1^. When the compaction velocity surpasses 5.6 ms^−1^, the sintered density continues to increase but at a much slower rate. It is worth mentioning that the sintered density is always higher than the green density, and there is no swelling during the sintering. The maximum sintered density was recorded as 2.85 gcm^−3^ (the relative density is 98%) when the compaction velocity was 9.4 ms^−1^.

### 3.3. Shrinkage

**Figure 7:**
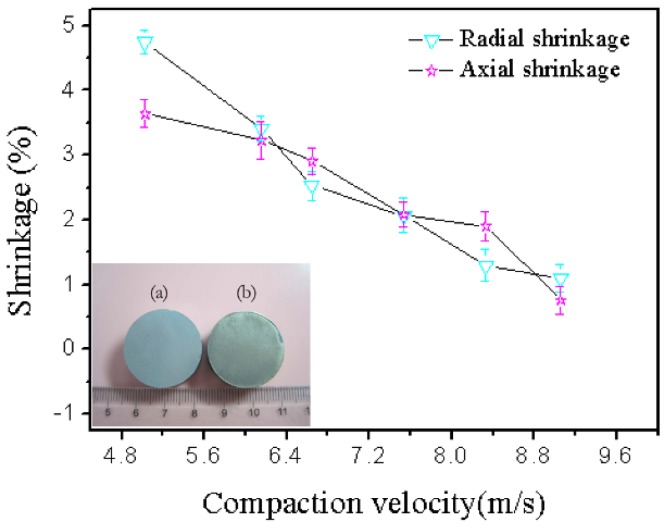
Shrinkage versus compaction velocity and cylindrical. Shapes of the green (**a**) and sintered (**b**) samples.

Sintering is driven by the elimination of surface energy. The sintering of the green components induces a reduction of their dimension and, therefore, the samples undergo shrinkage [32]. The bulk transport process changes the compact density by removing mass from the contacts between the particles and redepositing that mass to form the neck. The result is compact powder shrinkage [33]. Sintering generally leads to shrinkage of the macroscopic size of the sample, and the shrinkage rate can be used to measure the degree of sintering. Sintering shrinkage is due to sintering neck growth and is caused by shortening the distance between the powder particles. This shrinkage is the compact dimension change divided by the initial dimension. The radial shrinkage is the change of the radial dimension of the sintered samples, and the axial shrinkage is the change of the axial dimension of the sintered samples. The radial and axial shrinkages were tested, and the shrinkage δ was calculated using Equation (6):

(6) $$\delta =\frac{\Delta l}{l_{0}}\times 100\%=\frac{l_{0}-l}{l_{0}}\times 100\%$$

where *l*_0_ is the radial or axial length of the sample before sintering (mm), and *l* is the radial or axial length of the sample after sintering (mm). Figure 7 demonstrates that the shrinkage decreases with increasing compaction velocity. Cooke also demonstrated that all bars of the PM 2618 aluminum alloy exhibited appreciable amounts of shrinkage in each of the three principal directions, and the net amount of shrinkage was reduced as the compaction pressure increased [33]. This was because the higher compaction velocity produced higher compaction pressure, which was explained by Equation (7). Higher compaction pressure was helpful in decreasing the porosity, and distance between particles. Accordingly, $l_{0}-l$ was reduced and the shrinkage diminished. The result of the shrinkage was in agreement with the sintered density:

F = MV/t(7)

where F is the impact force, kN; M is the mass of the hammer and the upper punch, kg; and t is the time, s.

During HVC, the upper die and hammer with a high velocity and mass (which generates high impact force within 200 ms) impact the powder, and then the powder is compressed by the impact force. The compression process is divided into two stages. In the first stage, compact densification occurs mainly in the form of the displacement and rearrangement of particles to eliminate the large pores. In the second stage, the kinetic energy acting on the powder body dissipates to slide, rotate, deform, and break particles, [25]. During the second stage, with an increase of impact velocity, the hammer and upper punch further compress the powder, and the powder particles are subjected to axial direction impact force beyond the limit of the hardness of the powder, and powder particles experience deformation, which causes a radial direction expansion of the powder particles. Thus, the radial direction gap is squeezed, and then further extension of the particles in the radial direction is prevented by the die wall, which makes the particles’ radial sizes constant. In other words, the die wall produces lateral pressure on the powder. Therefore, with the combined action of the powder particles in the axial direction and the impact force and lateral pressure, the contact area between the particles increased and experienced densification. Sintering the green sample, because of their large contact area, produced large surface energy, which was the driving force of sintering. The sintered samples obtained a high density and low shrinkage rate, and change in sample size was small when the compaction velocity was high.

### 3.4. Tensile Property

**Figure 8:**
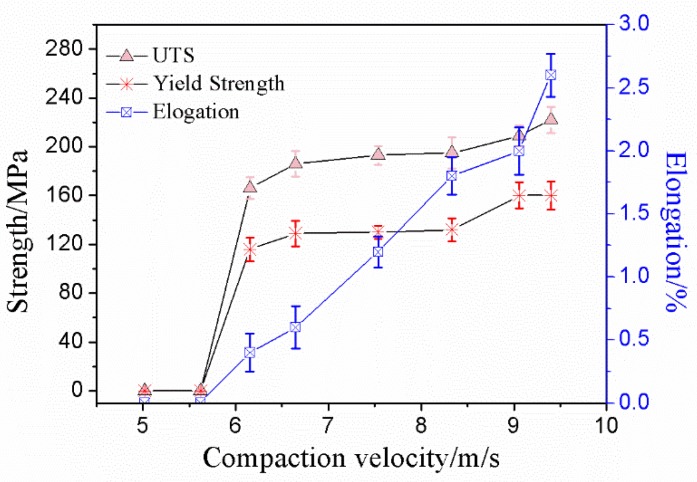
Ultimate tensile strength, yield strength and elongation versus compaction velocity.

The tensile property of the sintered compacts was measured by using a flat dog bone style tensile specimen. The data of the ultimate tensile strength (UTS), 0.2% yield strength (YS), and elongation are shown in Figure 8. It can be seen from Figure 8 that the ultimate tensile strength and 0.2% yield strength both increased by increasing the compaction velocity, which reached its maximum (UTS 222 MPa, YS 160 MPa) at a compaction velocity of 9.4 ms^−1^, and the maximum elongation was 2.6%.

When the compaction velocity was less than 5.6 ms^−1^, UTS, YS, and elongation of the sintered samples were zero. A lower compaction velocity generated a lower impact force for the hammer on the powder, which resulted in a lower bending strength for the green material. In the previous work, the bending strength of the green material was 7.3 MPa when the compaction velocity was 5.6 ms^−1^ [26]. The bond strength of green material is very low, and, therefore, the ultimate tensile strength, yield strength, and elongation of the sintered samples were zero. When the compaction velocity was from 5.6 ms^−1^ to 6.2 ms^−1^, the UTS and YS of the sintered compacts increased quickly, and increments were 166 Mpa and 116 MPa, respectively. When the compaction velocity was from 6.2 ms^−1^ to 7.9 ms^−1^, the UTS and YS of the sintered compacts increased slowly, and increments were 9 Mpa and 3 MPa, respectively. When the compaction velocity was from 7.9 ms^−1^ to 9.4 ms^−1^, the UTS and YS of the sintered compacts increased quickly, and increments were 27 Mpa and 28 MPa, respectively. The elongation increased nearly linearly with increasing the compaction velocity.

**Figure 9:**
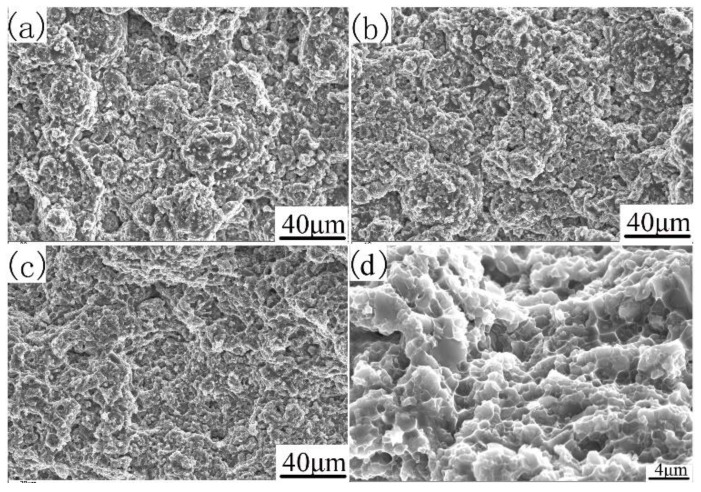
SEM/SE of the fracture surface of sintered samples. With different compaction velocities (**a**) 6.2 ms^−1^, (**b**) 7.9 ms^−1^, (**c**) and (**d**) 9.4 ms^−1.^

The fracture morphology can be further explained, as shown in Figure 9. The tensile fracture surfaces of the investigated alloys with different compaction velocities are shown in Figure 9. The transitions in tensile ductility among the specimens with different compaction velocities are accompanied by obvious changes in the fractures’ surface appearance. It can be seen from Figure 9a that the sample with a compaction velocity of 6.2 ms^−1^ has obvious particle boundaries, and there are some dimple formations on the particle’s surface. These particle boundaries cause the degradation of mechanical properties. In the sample with a compaction velocity of 7.9 ms^−1^ (as shown in Figure 9b), prior particle boundaries began to disappear, and dimple formations began to appear. These dimple formations are characteristics of particle bonding. In the sample with a compaction velocity of 9.4 ms^−1^ (as shown in Figure 9c), prior particle boundaries disappeared. The sample reveals an obvious presence of dimples, thereby supporting the occurrence of inter-particle bonding [34]. Figure 9d shows the amplifying fracture morphology of Figure 9c. As shown in Figure 9d, deep and small dimples were found everywhere on the fracture surface of the sintered sample with a compaction velocity of 9.4 ms^−1^, indicating that the fracture mechanism is a ductility fracture. In addition, the rupture of the oxide film contributed to the particle boundary strengthening.

## 4. Discussion

During the HVC, the compaction force increased alongside an increase in the compaction velocity [26]. The green density and sintered density also increased with increasing compaction velocity, which was in agreement with the results reported by Showaiter [10]. Showaiter reported that by increasing the compaction pressure, the sintered density increased, because of greater particle bonding and the presence of fewer voids as the compaction pressure increased. In contrast, Heard [35] showed that pressing with excess loads resulted in an even denser green component, yet the sintered product was mildly inferior to a product compacted at a lower pressure. It was postulated that the reduced amount of interconnected porosity might result in a retardation of the removal of the binder additive. However, the Al-based powder compacted by HVC did not contain a binder, and there was only die-wall lubrication before the hammer impacting. Lubricants cannot be mixed with the powder since they take up too much volume, so only die-wall lubrication is applicable [36]. Because of this, it was reasonable for HVC that the higher green density yielded a higher sintered density. Thus, the high green density obtained ensures good green strength, which can aid with ejecting the compacts from the die cavity and their subsequent handing prior to sintering. These characteristics are vital to the net shape capability of the Al-based PM alloy. Overall, increasing the compaction velocity is beneficial for the sintered density of this Al-based alloy.

The present investigation of an Al–Fe–Cr–Ti alloy prepared by HVC followed by sintering provides a detailed account of the alloy’s microstructure evolution and phase formation. Most importantly, this study reports the relationship between the compaction velocity of HVC and sinterability. In addition, it also provides an account of the mechanical properties measured using tensile testing. We discuss the results of the investigation in the following: (1) The microstructure and phase constitution of the sintered sample; and (2) the strengthening mechanisms.

### 4.1. Microstructure and Phase Constitution of the Sintered Sample

**Figure 10:**
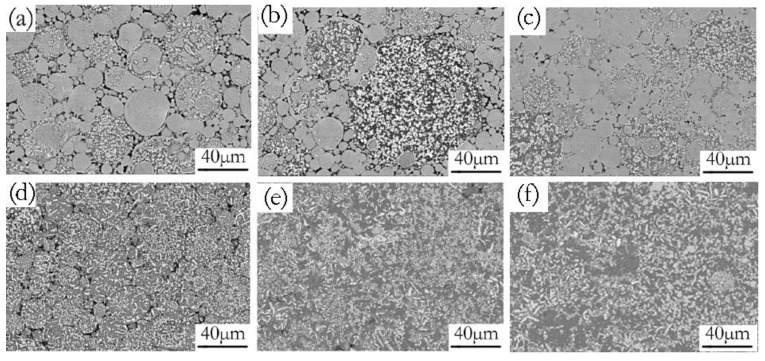
SEM/BSE micrographs of green (**a**–**c**) and sintered (**d**–**f**) samples under the different compaction velocities (a,d) 6.2 ms^−1^, (b,e) 7.9 ms^−1^, (c,f) 9.4 ms^−1^.

Increasing the green density promoted more intimate contact between the adjacent powder particles. The increased particle–particle contact area would then facilitate densification upon sintering because of the improved diffusion pathways and narrower spacing between particles [37]. An illustration of the effects of compaction velocity on the sintering response is given in Figure 10. Figure 10a–c are SEM micrographs of green samples pressed by a velocity of 6.2 ms^−1^, 7.9 ms^−1^, and 9.4 ms^−1^. Figure 10d–f are the SEM micrograph of the sintered samples pressed by a velocity of 6.2 ms^−1^, 7.9 ms^−1^, and 9.4 ms^−1^. Figure 10a exhibits the green material with a compaction velocity of 6.2 ms^−1^, which has obvious particle boundaries and pores. Accordingly, there are still obvious particle boundaries and pores when the green material is sintered, as shown in Figure 10d. Figure 10b shows that the green material with a compaction velocity of 7.9 ms^−1^ still has particle boundaries and pores, but the contact area between the particles increases. For the sintered sample, there are no obvious particle boundaries, but it has a few pores, as shown in Figure 10e. Figure 10c shows that the number of pores in the green material with a compaction velocity of 9.4 ms^−1^ decrease, and the contact area between the particles increases. Accordingly, the complete dissolution of the particles and the elimination of porosity for the sintered sample can be observed in Figure 10f. Figure 10d–f illustrate that a higher compaction velocity also contributes to a larger sintered neck size. The sintered neck size determines the properties such as strength and ductility. During HVC, the contact area between the green particles and the inter particles’ bonding strength increased as the compaction velocity increased, which was helpful for enhancing sinterability [38].

**Figure 11:**
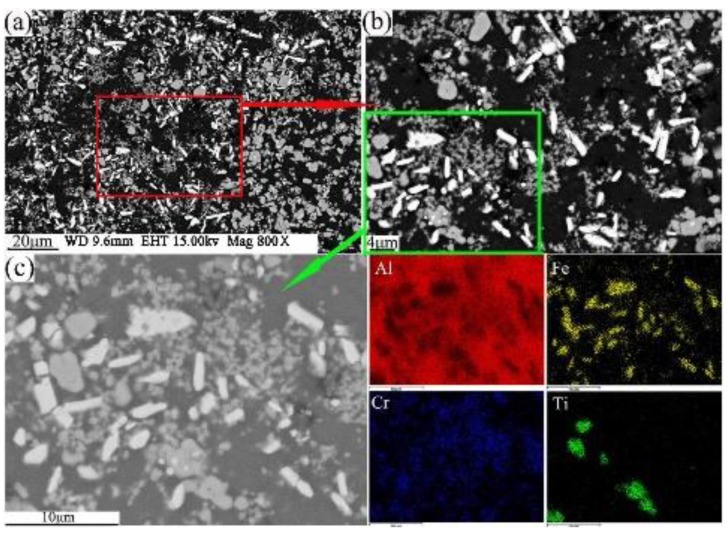
FESEM/BSE morphology of sintered sample with compaction velocity 9.4 ms^−^^1^ (**a**,**b**); Al, Fe, Cr, and Ti element EDS mapping (**c**).

**Figure 12:**
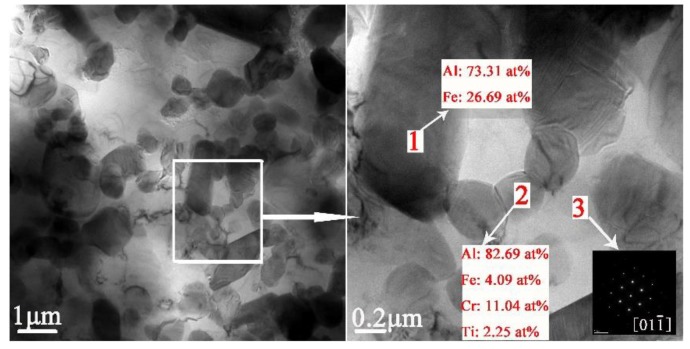
TEM/BF micrograph of sintered sample with compaction velocity 9.4 ms^−^^1^: bright field images for intermetallic compound.

The present study has clearly demonstrated that the phases formed in the green material undergo a transformation during sintering. It is obvious that sintering has a strong influence on the phase formation. XRD patterns of the sintered sample are shown in Figure 5b. These patterns reveal that the phases of the sintered alloy mainly consisted of Al_13_Fe_4_, Al_13_Cr_2_, AlCr_2_, Al_3_Ti, and AlN. Among these phases, Al_13_Fe_4_ and Al_13_Cr_2_ are produced by the decomposition of the Q-phase in fine particle powders [39]. Pedrazzini et al. [40] and García et al. [39] reported the phases of Al-Fe-Cr-Ti alloy. They thought the phases of this alloy were consist with Al_13_Cr_2_, Al_13_Fe_4_, and Al_3_Ti. These results are consistent with our experiments. Vojtech et al. [18] demonstrated the decomposition reaction, as shown in Equation (8). Figure 11 displays the microstructure and the EDS mapping results of the sintered samples of the Al, Fe, Cr, and Ti elements. Figure 11a presents the white needle phases and gray flake phases. The red frame of Figure 11a is further amplified, as shown in Figure 11b. Figure 11b shows a dispersion of small particles. Figure 11c shows the green frame of Figure 11b. The EDS mapping result of Figure 11c shows that the white needle phases are rich in Fe, and the gray flake phases are rich in Ti. The small particles are rich in Cr, which disperse and distribute in the Al matrix. This result is further confirmed by TEM, as shown in Figure 12, where the presence of the spherical small particle phase and the rod phase can be seen. Combined with the EDX analysis results, the higher Cr content of phase 2 is presumed to be Al_13_Cr_2_, and the higher Fe content of phase 1 is presumed to be Al_13_Fe_4_. Phase 3 is the α-aluminum matrix. Al_3_Ti is a high melting point compound with good thermal stability [41]. Al_13_Cr_2_ hinders the nucleation and growth process of recrystallization, which has a strengthening effect on the alloy and can also improve the toughness of the alloy, thereby reducing the sensitivity of the stress corrosion cracking [42]. AlN is a product of an aluminum alloy that reacts with a protective atmosphere of nitrogen during sintering. This reaction is shown in Equation (9) [43]. This is an exothermic reaction, in which $\Delta_{f}H_{\mathrm{AlN}}^{0}$ is −318 KJ/mol, and the heat generated by the reaction can locally melt the aluminum alloy and promote sintering. Schaffer et al. [12] also found that this reaction reduced the pore pressure in the pore spaces relative to the external atmosphere, which induced pore filling at grain sizes smaller than those required for sintering in an inert atmosphere. Pore filling is an important densification mechanism during the sintering of aluminum:

(8) $$Q-\mathrm{phase}\to \mathrm{fcc}-{\mathrm{Al}}_{13}{\mathrm{Cr}}_{2}+{\mathrm{Al}}_{13}{\mathrm{Fe}}_{4}$$

(9) $$2\mathrm{Al}+N_{2}\to 2\mathrm{AlN}$$

### 4.2. Strengthening Mechanisms

**Figure 13:**
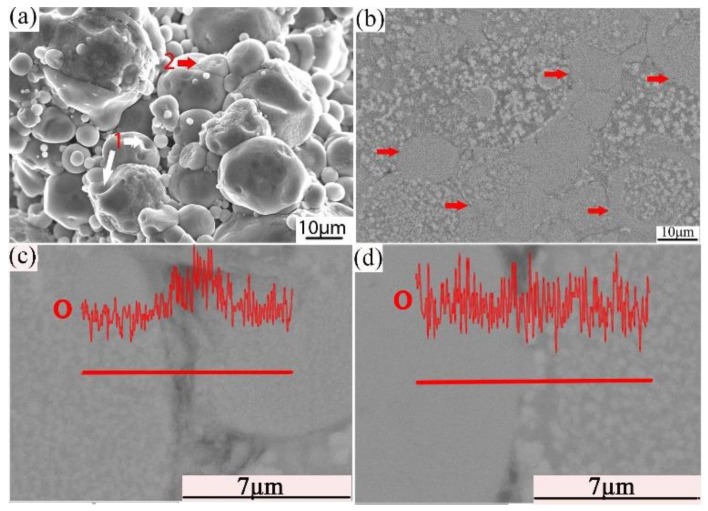
SEM/SE micrograph of the fracture surface (**a**) cross section (**b**); EDS line scan of O in the particle boundary (**c**); and EDS line scan of O in the deformed particle boundary (**d**) of the green with a compaction velocity of 9.4 ms^−1.^

Combined with the microstructure and phase constitution analysis, the enhanced tensile properties of the Al–Fe–Cr–Ti alloy are due to the following strengthening mechanisms: (1) Particle boundary strengthening, (2) intermetallic compound strengthening, and (3) solid solution strengthening. Among these, particle boundary strengthening is the main strengthening mechanism. Ultimate tensile strength, yield strength, and elongation increased by increasing the compaction velocity. Increasing the compaction velocity facilitated the deformation and bonding of the particles. Accordingly, an increased particle–particle contact area facilitated densification upon sintering because of improved diffusion pathways and narrower spacing between the particles. Hence, the strength of the alloy was increased [44]. The HVC process provided a mechanical means of disrupting the oxide film because of the considerable distortion of the ductile aluminum powder that occurred during the processing [45]. The impact force upon the particles increased as the compaction velocity increased. The high impact force broke the oxide films coated on the surface of the aluminum alloy particles. These particles formed genuine metal-to-metal contacts that facilitated diffusion and caused the bonding force between the particles to increase [34,36]. Figure 13a shows an SEM micrograph of the fracture surface of the green material with a compaction velocity of 9.4 ms^−1^. With a high impact force, the small particles were pressed into the big particles. The surface of the big particles leaves many holes, as shown by white arrow 1 in Figure 13a. Half of the small particle was pressed into the big particle, as shown by red arrow 2 in Figure 13a. Figure 13b displays a highly magnified SEM image of the green material with a compaction velocity of 9.4 ms^−1^. As observed in Figure 13b, the contact between the particles is intact; not only are there no pores, but one can also see the phenomenon of the extrusion deformation of the particle boundary, as shown by the red arrows. In the process of the extrusion deformation of the particle boundary, the complete oxide film is broken. Figure 13c,d show the oxygen content at the complete particle boundaries is obviously higher than that inside the particles. Oxygen content is low at the boundaries of the deformed particles, which shows that the oxide film is interrupted at the particle boundaries. A local fracture of the large particle oxide film is helpful for sintering [6]. The general understanding is that the oxide film is ruptured at points where the particles touch during powder compaction to form genuine metal-to-metal contacts. Accordingly, genuine metal-to-metal contacts facilitate diffusion of the alloy element and improve the sintered strength. In addition, the broken oxide film can also act as a particle enhancement [46], thereby increasing the strength. On the other hand, intermetallic compound strengthening is also obvious. Some strengthening effects may be associated with fine intermetallic particles, such as Al_13_Cr_2_. From Figure 11c and the EDS mapping of Cr it can be observed that Al_13_Cr_2_ particles disperse in the Al matrix. From Figure 12 it is observed that the Al_13_Cr_2_ particles size is in the range of 200–500 nm. Fine Al_13_Cr_2_ hinders dislocation mobility, thereby augmenting the yield strength [47]. Finally, a solid solution mechanism was found to play a minor role because of the sintering method used [47].

The strength is lower than 600 MPa for the Al–Fe–Cr–Ti alloy in the literature [48]. The main areas of study for this analysis are as follows: (1) The growth of the intermetallic phase, such as Al_13_Fe_4_; and (2) decomposition of the quasicrystal phase. The decomposition of the Q-phase during sintering has a detrimental effect on the strength of the Al–Fe–Cr–Ti alloy [39]. These problems are related to the higher sintering temperature, and we will continue to study the sintering process in depth.

## 5. Conclusions

The work completed in this study has led to the following conclusions:

(1) The rapid impact of high velocity compaction not only contributes to an increased green density but also contributes to an increased sintered density. A maximum sintered density of 2.85 gcm^−3^ (relative density is 98%) was obtained when the compaction velocity was 9.4 ms^−1^.

(2) Shrinkage decreased by increasing the compaction velocity. The shrinkage was rather isotropic, as the linear contractions along the two directions were very similar. The radial and axial shrinkage could be controlled to less than 1% at the compaction velocity of 9.4 ms^−1^.

(3) At a compaction velocity of 9.4 ms^−1^, sintered compacts with an ultimate tensile strength of 222 MPa and a yield strength of 160 MPa were achieved. The maximum elongation was observed at 2.6%.

(4) The enhanced tensile properties of the Al–Fe–Cr–Ti alloy are due to particle boundary strengthening. In addition, intermetallic compounds, such as fine Al_13_Cr_2_, augments the yield strength. The effects of solid solution strengthening, however, are not obvious.

## Figures and Tables

**Table 1:** Composition (in wt%) of the aluminum alloy powder.

Element	Al	Fe	Cr	Ti	Si	Zn	Cu	Ca	Ni	Ga
Wt%	balance	5.79	3.56	3.32	0.08	0.03	0.02	0.02	0.02	0.01

**Table 2:** Relationship between stroke length and velocity.

Stroke Length/mm	20	25	30	35	40	45	50	55	60	65	70
velocity/m/s	5.0	5.6	6.2	6.7	7.1	7.5	7.9	8.3	8.7	9.1	9.4

**Table 3:** The energy dispersive spectroscopy (EDS) of the three phases of the arrow in the microstructures of the particles.

Element	Phase	Al	Ti	Cr	Fe
Atomic%	1	80.34	0.55	5.95	13.15
Atomic%	2	78.75	20.02	0.30	0.94
Atomic%	3	87.37	3.24	3.72	5.67
